# Response to the letter to the editor: Clomiphene or enclomiphene citrate for the treatment of male hypogonadism: a systematic review and meta-analysis of randomized controlled trials

**DOI:** 10.20945/2359-4292-2025-0547

**Published:** 2025-11-22

**Authors:** Alexandre Hohl, Matheus Pedrotti Chavez, Eric Pasqualotto, Rafael Oliva Morgado Ferreira, Simone van de Sande-Lee, Marcelo Fernando Ronsoni

**Affiliations:** 1 Departamento de Clínica Médica, Universidade Federal de Santa Catarina, Florianópolis, SC, Brasil

Dear Editor,

We thank Dr. Bo Peng for the thoughtful and constructive comments on our paper “Clomiphene or Enclomiphene Citrate for the Treatment of Male Hypogonadism: A Systematic Review and Meta-Analysis of Randomized Controlled Trials” (^1^). His observations allow us to clarify several methodological and conceptual points.

## 1. CLINICAL HETEROGENEITY AND TARGET POPULATION

We acknowledge that the included randomized controlled trials (RCTs) involved patients with differing metabolic phenotypes and baseline testosterone levels. To address this, our protocol prespecified inclusion only of adults with functional or secondary hypogonadism (total testosterone ≤ 300 ng/dL) and excluded primary forms (^2^). Subgroup and meta-regression analyses were conducted to explore sources of variability, revealing that age and body mass index (BMI) partially explained the heterogeneity of testosterone response. Despite differences in study populations, the direction of effect was consistent, supporting a generalizable increase in endogenous testosterone with both clomiphene and enclomiphene (^1^). Distinguishing functional from organic hypogonadism was a central premise of our analysis, as emphasized in the Discussion section.

## 2. BIOCHEMICAL VERSUS PATIENT-CENTERED OUTCOMES

We agree that symptomatic endpoints and quality-of-life measures deserve greater attention in future research. Unfortunately, most available RCTs reported only biochemical variables, limiting quantitative synthesis of validated scales such as International Index of Erectile Function (IIEF) or Androgen Deficiency in Aging Males (ADAM). In our review, patient-reported outcomes were therefore described narratively, and we highlighted this limitation. We fully support defining clinically meaningful thresholds for symptom improvement and metabolic or bone health markers in upcoming trials (^3^).

## 3. FERTILITY ENDPOINTS AND REPRODUCTIVE RELEVANCE

As noted, selective estrogen receptor modulator (SERM) therapy was superior to testosterone gel for sperm parameters, consistent with restoration of hypothalamic-pituitary-gonadal axis activity (^1^). However, pregnancy and live-birth outcomes were not available from existing RCTs. We explicitly stated that these findings should be interpreted as potential fertility-preserving effects rather than definitive evidence of improved reproductive success (^4^,^5^). We concur that future studies should evaluate conception and live-birth rates and compare SERMs, human chorionic gonadotropin (hCG), and combination regimens.

## 4. OVERALL INTERPRETATION

Our conclusions were framed within the limits of available data: SERMs effectively raise total testosterone, luteinizing hormone (LH), and follicle-stimulating hormone (FSH) in men with functional hypogonadism and represent a physiological, fertility-preserving alternative to exogenous testosterone (^1^,^4^). The commentary reinforces the need for more comprehensive, long-term, and patient-centered randomized trials to translate these biochemical improvements into tangible clinical benefit (^3^).

We appreciate the opportunity to clarify these points and the valuable dialogue that advances evidence-based care for men with hypogonadism.

## Data Availability

datasets related to this article will be available upon request to the corresponding author.
